# Prevention of contrast-associated acute kidney injury in an era of increasingly complex interventional procedures

**DOI:** 10.3389/fmed.2023.1180861

**Published:** 2024-01-09

**Authors:** Cristina Somkereki, Renata Palfi, Alina Scridon

**Affiliations:** ^1^Cardiology Department, Emergency Institute for Cardiovascular Diseases and Transplantation Târgu Mureș, Târgu Mureș, Romania; ^2^Physiology Department, University of Medicine, Pharmacy, Science and Technology “George Emil Palade” of Târgu Mureș, Târgu Mureș, Romania

**Keywords:** contrast-associated acute kidney injury, percutaneous coronary intervention, periprocedural fluid administration, prevention, risk prediction

## Abstract

Radiological and interventional cardiology procedures are in continuous expansion, leading to an important increase in the incidence of contrast-associated acute kidney injury (CA-AKI). Although numerous methods of CA-AKI prevention have been studied, at present, there is no consensus on the definition of this entity or on its prevention. In this paper, we aim to provide a critical analysis of the existing data on the epidemiology, pathophysiology, and clinical significance of CA-AKI. Existing and emergent approaches for CA-AKI prevention are also discussed, with a focus on parenteral fluid administration and on the most recent clinical and experimental data. We also emphasize a number of questions that remain to be answered, and we identify hotspots for future research.

## 1 Introduction

For decades, physicians have been concerned about the increasing use of contrast agents and their risk of inducing acute kidney injury (1). Despite substantial research, there are still many unknown aspects related to contrast-associated acute kidney injury (CA-AKI). Consensus appears to exist among physicians that contrast-induced complications are not clinically significant, and some authors question the very existence of this entity (2). Meanwhile, numerous clinical studies support a deleterious effect of contrast agents on kidney function, and animal studies clearly showed harmful effects (3–7).

The use of less nephrotoxic substances has reduced the incidence of CA-AKI (8). However, at this point, there is no consensus on the exact incidence of CA-AKI in the era of modern contrast agents, nor on the medium- and long-term impact of CA-AKI. Also, a wide array of strategies with prophylactic and/or therapeutic potential have been evaluated, but there is no consensus on the most effective strategy. Data regarding preventive measures such as fluid administration, pharmacological strategies, use of different contrast agents, and renal replacement therapies are conflicting. Isotonic saline administration seems to be, however, the most effective way to prevent CA-AKI (9–11). In addition, although many factors have been studied, with the exception of basal renal function, there is no consensus on the factors that increase the risk of CA-AKI.

In this paper, we aim to provide a critical analysis of the existing data on the epidemiology, pathophysiology, and clinical significance of CA-AKI. Existing and emergent approaches for CA-AKI prevention are also discussed, with a focus on parenteral hydration and on the most recent clinical and experimental data in this field.

## 2 Epidemiology of contrast-associated acute kidney injury in the era of modern iodinated contrast media

In a recent study including more than 7,000 patients, CA-AKI occurred in 6.5% of patients receiving intra-arterial administration of contrast media (12). In other studies, intra-arterial contrast administration led to CA-AKI in more than 13% of patients, whereas the risk of CA-AKI following intravenous contrast administration appeared to be negligeable (13). Development of CA-AKI after cardiac and radiologic procedures is associated with a significant increase in morbidity, mortality, and costs, and with prolonged hospitalization (14). At present, CA-AKI is defined as an impairment of renal function after parenteral administration of contrast media in the absence of other identifiable causes, characterized by an increase in serum creatinine (SCr) of at least 0.5 mg/dL (≥44 μmol/L) or ≥ 25% above baseline within the first 48 h after contrast administration (14). This is also the definition most frequently used in clinical trials. The European Society of Urogenital Radiology defines CA-AKI based on an increase in SCr by ≥0.5 mg/dL or ≥ 25% within the first 3 days after intravascular administration of contrast medium, without an alternative etiology (15).

The incidence of CA-AKI is increasing mainly due to the increase in the number of interventional procedures. In the United States only, over one million cardiac catheterization procedures are performed annually (16). The continuous development of interventional cardiology has also led to increasingly complex procedures that require administration of considerably larger amounts of contrast material (17). According to the study by Jennings et al., the crude rate of angiography and percutaneous coronary interventions (PCI) performed in Ireland increased by 47.8 and 35.9%, respectively, from 2004 to 2011, with rates of 6,689 and 1,825 per million individuals in 2011 (17). Among those cases, the reported incidence of CA-AKI ranged from 0 to >50% (18–21). Similar increases in the number and complexity of interventional cardiology procedures were also seen in more recent studies performed in Spain and Switzerland (22, 23).

A recent review and meta-analysis pointed out a higher incidence of CA-AKI after intra-arterial than after intravenous contrast medium administration, but the overall incidence of CA-AKI seemed to be decreasing over time, together with the need for dialysis. The incidence of CA-AKI was 9% and that of kidney failure requiring dialysis was 0.5% after intra-arterial administration of contrast medium (24). According to Chalikias et al. (25), the incidence of CA-AKI after PCI ranges from 2 to 20%, depending on baseline kidney function.

In addition to the increasing number of angiographies and computed tomography (CT) examinations, causing an increasingly number of patients to be exposed to contrast media, the population exposed to these procedures is also older and sicker, and the volume of contrast media administered is also higher in these patients (26–31).

The incidence of CA-AKI also seems to be influenced by several other major factors, including the type, volume, and route (arterial vs. venous) of contrast media administered. While there is agreement that intra-arterial contrast agent administration can cause acute kidney injury and that the incidence of CA-AKI is significantly higher after intra-arterial than after intravenous contrast administration, retrospective studies suggest that intravenous contrast material administration for CT examinations may associate a similar risk of CA-AKI to non-contrast CT (32, 33). Meanwhile, according to a systematic review and meta-analysis published in 2016, CA-AKI risk did not differ among types of low-osmolar contrast media and there was also no difference between the route of administration of the contrast media (34).

## 3 Brief overview of the pathophysiology of contrast-associated acute kidney injury

**Figure 1:**
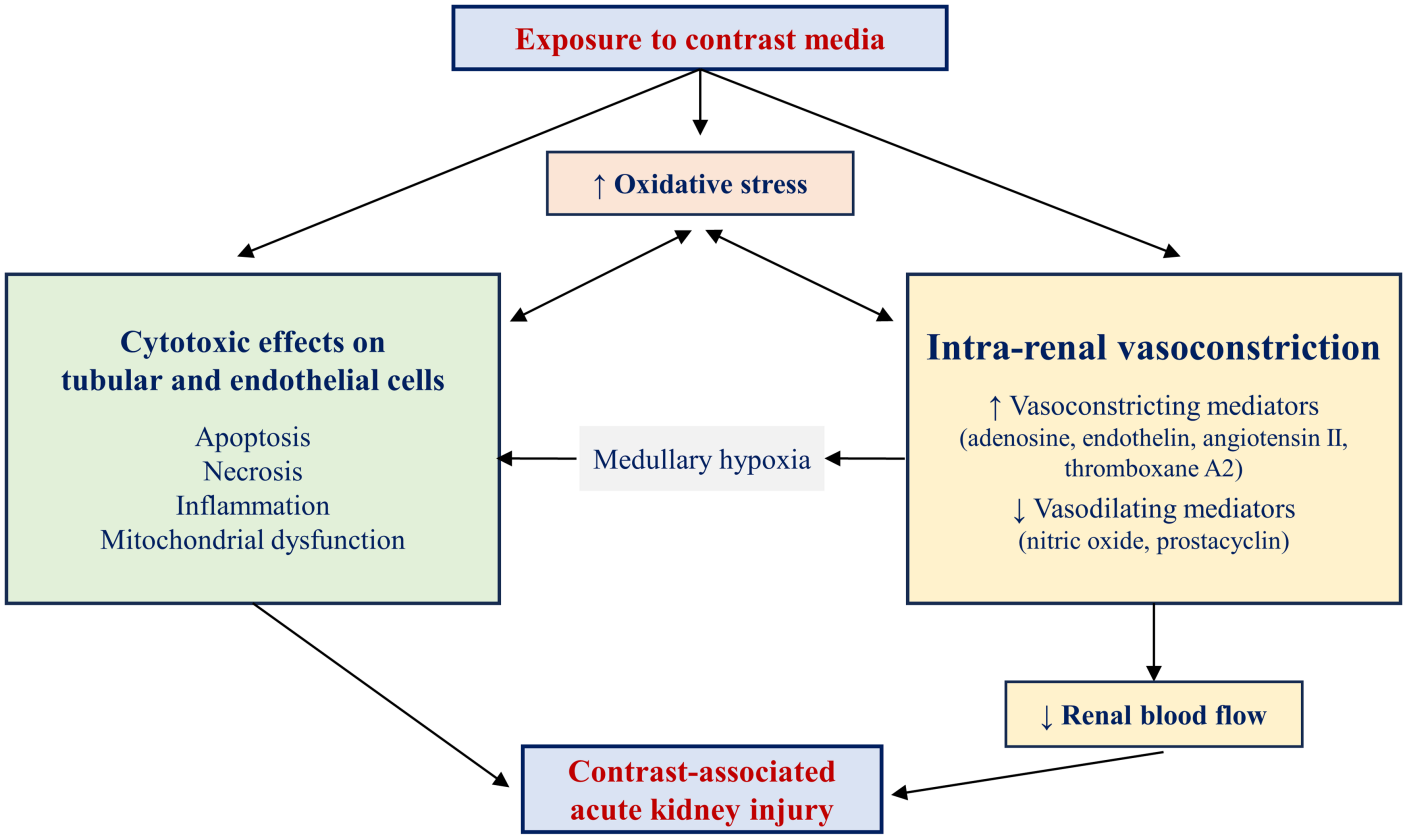
Pathophysiology of contrast-associated acute kidney injury. Two main mechanisms contribute to the pathogenesis of contrast-associated acute kidney injury: direct cytotoxic effects on tubular and endothelial cells and intrarenal vasoconstriction, favored by an imbalance between vasoconstricting and vasodilating mediators. The medullary hypoxia resulting from renal vasoconstriction further alters the structure and function of renal tubular cells. The increased production of free oxygen species and the consequent increase in oxidative stress aggravate renal vasoconstriction and exert toxic effects on renal tubular cells. In parallel, renal ischemia and local cytotoxic effects aggravate oxidative stress, creating a vicious cycle. The coexistence and mutual stimulation of these mechanisms eventually result in contrast-associated acute kidney injury.

The exact mechanisms of CA-AKI remain to date unknown. Several causes have been described and there are two major theories regarding the mechanism of CA-AKI: direct cytotoxic effects of contrast media and renal vasoconstriction resulting in medullary hypoxemia (Figure 1) (21). Contrast agents have been shown to exhibit direct toxic effects on renal tubular cells, caused by oxygen free radicals release, altered mitochondrial function, and apoptosis, all culminating in renal ischemia (35, 36). Contrast agents exert their toxic effects mainly on renal tubular epithelial cells, causing vacuolization and osmotic nephrosis, and on endothelial cells. Although the exact mechanisms responsible for contrast media-induced cytotoxicity remain elusive, these agents have been shown to stimulate cellular pathways involved in apoptosis by activating caspase-3, caspase-9, and the bcl2 pathway (37). In addition, administration of contrast media has been linked to redistribution of cell membrane proteins and disruption of intercellular junctions, DNA damage, mitochondrial dysfunction, and a significant reduction in cell proliferation, leading to alterations in cell polarity, loss of cytoskeletal integrity, displacement of membrane proteins and cellular adhesion molecules, and necrosis of tubular epithelial cells (38–42).

The toxicity of contrast media on the tubular and endothelial cells is further accentuated by oxidative stress and hypoxia (43, 44). Increased adenosine and endothelin release in response to contrast agent administration leads to renal vasoconstriction, whereas reduced nitric oxide and prostaglandin synthesis decreases the vasodilation of the renal vascular bed (36). Together, these mechanisms are responsible for ischemia in the deeper portion of the outer medulla, an area with increased oxygen demand. Many of the deleterious effects of contrast agents on the kidneys are mediated by increased production of oxygen free radicals and oxidative stress. Medullary hypoxia and adenosine catabolism increase the production of oxygen free radicals, which not only scavenge nitric oxide, but also increase the synthesis of vasoconstricting substances such as endothelin, adenosine, angiotensin II, and thromboxane A2. Constriction of glomerular and peritubular capillaries and of *vasa recta* further leads to alterations in renal perfusion and blood flow autoregulation, and to distal ischemia (45, 46). At its turn, ischemia then increases the formation of oxygen free radicals, thus leading to a vicious circle in which ischemia promotes oxidative stress and oxidative stress aggravates the preexisting ischemia. Experiments performed on isolated mouse kidney vessels showed that decreased nitric oxide availability and increased superoxide production induced by contrast media resulted in more pronounced vasoconstriction in the afferent than in the efferent arterioles, providing a pathophysiological explanation for the reduction in glomerular filtration following administration of contrast media (47). In addition, increased oxidants levels alter mitochondrial and nuclear DNA, lead to damage in membrane lipids and cellular proteins, and activate c-Jun N-terminal, ERK, and p38-MAPK kinases, promote apoptosis and necrosis via activation of caspase-3 and caspase-9 (48, 49).

## 4 Clinical significance of contrast-associated acute kidney injury

### 4.1 Predictors of contrast-associated acute kidney injury

While in the general population the incidence of CA-AKI has been estimated to be <2%, in patients at high risk (patients with diabetes mellitus [DM], congestive heart failure, chronic renal impairment, and older age) the incidence is estimated to be >20% and up to 30% (50). In 2008, Mehran et al. (14) proposed a risk score for prediction of CA-AKI after PCI. In this score, points are given for the presence of hypotension, use of intra-aortic balloon pump, symptoms of congestive heart failure, age, anemia, DM, volume of contrast media administered, and the estimated glomerular filtration rate (eGFR). According to the Mehran risk score, a score < 6 points indicates a risk for CA-AKI of 7.5%, a risk score between 6 points and 10 points indicates a risk for CA-AKI of 14%, a risk score between 11 points and 16 points indicates a risk for CA-AKI of 26%, and a risk score > 16 points indicates a risk for CA-AKI of 57% following intra-arterial administration of contrast media (14).

Many risk factors have been reported to increase the risk of CA-AKI, but few of them have been identified as independent predictors of CA-AKI. Barett et al. analyzed a series of clinical trials using multivariate analysis and identified baseline renal disease, heart failure, DM, and the dose of contrast media administered as predictors of increased risk of CA-AKI, with pre-existing renal disease being the most significant predictor of CA-AKI (51).

Data regarding the role of DM as a risk factor for CA-AKI remain controversial. In Mehran’s risk score, DM is included as an independent predictor of CA-AKI. However, few articles have evaluated risk score models among patients with DM undergoing PCI. Hyperglycemia is associated with an increase in oxygen-derived free radicals, which will lead to vasoconstriction, one of the most important mechanisms involved in the pathophysiology of CA-AKI (52–54). Yao and co-workers proposed a simple clinical identification tool for predicting contrast-induced nephropathy in patients with DM undergoing PCI. The score includes four predictive factors and demonstrated good discrimination and predictive ability for CA-AKI and clinical outcomes after PCI (52). The study population was divided into groups according to the risk score: low (5.9% risk of CA-AKI), moderate (32.9% risk of CA-AKI), and high (60% risk of CA-AKI) risk score (52). Oxidative stress and inflammation have also been incriminated in CA-AKI occurrence in patients with acute ST-segment elevation myocardial infarction (36). Malnutrition and baseline inflammatory status have also been recently proposed as risk factors for CA-AKI in patients undergoing PCI (55, 56).

### 4.2 Impact of contrast media on the risk of contrast-associated acute kidney injury

At present, contrast media are commonly divided into high-osmolar (osmolality in the range of 1,000 to 2,000 mOsm/kg), low-osmolar (osmolality in the range of 500 to 1,000 mOsm/kg), and iso-osmolar (osmolality in the range of 290 to 300 mOsm/kg) contrast media. The introduction of low- and iso-osmolar contrast media has determined a reduction in the incidence of CA-AKI following intra-arterial contrast administration, particularly in high-risk patients (57).

In 1989, Schwab and co-workers published the results of a randomized controlled trial comparing a high-osmolar (diatrizoate) with a low-osmolar (iopamidol) contrast agent in 443 patients undergoing cardiac catheterization. They were unable to demonstrate a difference in the incidence of nephrotoxicity between the two groups (8, 58). Two years later, Taliercio and co-workers demonstrated, however, that the use of low-osmolar contrast medium (iopamidol) was less nephrotoxic than that of high-osmolar contrast medium (diatrizoate) in high-risk patients undergoing cardiac angiography. These findings were strengthened by the prospective randomized trial by Rudnick et al., which demonstrated that in patients with DM alone or combined with preexisting renal insufficiency undergoing cardiac angiography, the risk of CA-AKI was significantly lower when low- and iso-osmolar contrast media were used (20). In the Nephrotoxicity in High-Risk Patients Study of Iso-Osmolar and Low-Osmolar Non-Ionic Contrast Media (NEPHRIC) study by Aspelin et al. the incidence of CA-AKI was much lower when iodixanol was used rather than a low-osmolar non-ionic medium in patients with DM and in patients with preexisting renal insufficiency undergoing angiography (8). In patients undergoing invasive cardiac procedures, isoosmolal and low-osmolality agents have shown a significant reduction in the risk of CA-AKI compared with high-osmolality agents (57). In that study, iodixanol (an iso-osmolar agent) was shown to have the lowest risk for CA-AKI in patients at high risk (patients with chronic kidney disease and diabetes) (57). Iodixanol was shown to be superior to low-osmolality agents in this subset of patients and in those with renal dialysis and is thus recommended by The National Kidney Foundation Kidney Disease Outcome Quality Initiative Guidelines (57).

The risk of CA-AKI also increases as contrast volume increases. Thus, the recommended contrast doses in patients with chronic kidney disease are <30 mL for diagnostic catheterization and < 100 mL if a PCI is planned (57). According to the European Society of Cardiology, there are three simple ways of calculating maximum contrast volume to reduce the risk of CA-AKI. For example, < 100 mL should be used if significant chronic kidney disease is present and PCI is planned, or the volume of contrast should be adjusted according to SCr and body weight (5 x kg/SCr) or by multiplying 4 times the eGFR (calculated using the Cockroft-Gault or the Modification in Diet in Renal Disease equations) (57). According to recent data, use of systems specifically designed to reduce the volume of contrast media administered could further reduce risk of CA-AKI (59).

### 4.3 Clinical impact of contrast-associated acute kidney injury

#### 4.3.1 Short term implications of contrast-associated acute kidney injury

Contrast-associated acute kidney injury is associated with prolonged hospitalization and increased hospital-related costs, higher morbidity, and increased short-term mortality (60–66), although emergent hemodialysis is rarely needed following administration of iodinated contrast media (67). There are consistent data regarding the prolongation of hospitalization in patients with CA-AKI, proving that the higher the creatinine values, the higher the number of days of hospitalization, which will increase at its turn the socio-economic burden for the medical systems (68). According to Rihal et al., patients who developed CA-AKI after coronary angiography and PCI also had markedly higher incidence of in-hospital mortality than those who did not develop CA-AKI (22% vs. 1.4%; *p* < 0.0001) (68). Another retrospective trial demonstrated that even a small (0.25 mg/dL to 0.5 mg/dL) increase in SCr was associated with an increase of in-hospital mortality (69). Meanwhile, in a secondary analysis of the PRESERVE cohort, underlying chronic kidney disease was associated with cardiovascular events by day 90 following angiography, but this was not the case for CA-AKI, most of which was stage 1 (70).

#### 4.3.2 Long-term implication of contrast-associated acute kidney injury

In addition to short-term complications, CA-AKI is also associated with long-term mortality. Altered hemodynamics is associated with increased risk of CA-AKI and higher mortality rates. However, according to Sun et al., in a group of 696 patients with acute myocardial infarction and left ventricular ejection fraction >40%, CA-AKI was an independent predictor of long-term mortality, regardless of hemodynamic abnormalities (71).

## 5 Prophylactic strategies for contrast-associated acute kidney injury

### 5.1 Periprocedural fluid administration

Many randomized clinical trials have studied different pharmacological agents to prevent CA-AKI. However, according to an update published in the e-journal of the European Society of Cardiology Council for Cardiology Practice, intravenous fluid volume loading with isotonic saline (normal saline 0.9%) is considered the single most important measure to prevent the occurrence of CA-AKI (57). Volume resuscitation with isotonic saline is particularly important in hypovolemic patients. Questions remain, however, regarding the most adequate fluid and the optimal route of fluid administration – oral or intravenous (72). Trivedi and co-workers evaluated 53 patients undergoing cardiac catheterization. The patients were randomized into two groups: one group, consisting of 27 patients, received intravenous normal saline for 24-h (1 mL/kg/h) beginning 12 h prior to catheterization, and the other group, consisting of 26 patients, was allowed unrestricted oral fluids. Ten out of the 53 subjects developed acute renal insufficiency. The incidence of acute renal insufficiency was significantly lower in the group with saline administration (1 out of 27) as compared to the group allowed unrestricted oral fluids (9 out of 26; *p* = 0.005 between the two groups; relative risk: 0.11; 95% confidence interval 0.015 to 0.790) (73).

A randomized comparison of two fluid regimens (isotonic [0.9%] vs. hypotonic [0.45%] saline) performed in 1,620 patients undergoing coronary angioplasty was published by Mueller and co-workers, demonstrating that isotonic saline administration was superior to hypotonic hydration in the prevention of contrast media-associated nephropathy. In the group with isotonic saline administration, 5 of 685 patients developed CA-AKI (0.7%; 95% confidence interval 0.1–1.4%) vs. 14 of 698 patients in the hypotonic hydration group (2%; 95% confidence interval 1.0–3.1%; *p* = 0.04 between groups) (74). In the POSEIDON trial, left ventricular end-diastolic pressure-guided volume expansion with isotonic saline was more efficient than the standard (1.5 mL/kg/h) saline administration protocol in preventing CA-AKI in patients undergoing cardiac catheterization (75). Forced diuresis with dopamine, furosemide, or mannitol have shown no benefit in the prevention of CA-AKI, and their use has even been described to be harmful in different studies (76).

Current guidelines recommend prophylactic isotonic saline administration pre- and post-arterial administration of iodinated contrast media for prevention of CA-AKI in high-risk patients, based on expert consensus. The recommended protocol is 1 mL/kg/h 12 h before and continued for 24 h after the procedure or 0.5 mL/kg/h if the left ventricular ejection fraction is less than or equal to 35% and in heart failure patients with New York Heart Association class >2 according to the ESC/EACTS Guidelines on Myocardial Revascularization published in 2018 (53).

The A Maastricht Contrast-Induced Nephropathy Guideline (AMACING) trial demonstrated that no isotonic saline administration was non-inferior to prophylactic saline administration among patients with chronic kidney disease (eGFR 45–59 mL/min/1.73m^2^) and diabetes or at least two other risk factors (anemia, cardiovascular disease, ongoing nonsteroidal anti-inflammatory drugs or diuretics usage, age > 75 years), or multiple myeloma or lymphoplasmocytic lymphoma with small chain proteinuria receiving intravascular (intra-arterial or intravenous) contrast. A total of 660 patients were enrolled in that study, with a follow-up period of 35 days; 43% of patients were older than 75 years, 41% were females, 32% were diabetic, and all patients received the same low-osmolar contrast medium (iopromide). In patients with prophylactic isotonic saline administration, the incidence of symptomatic heart failure episodes was significantly higher than in the group with no saline administration. There was no difference between the two groups in the incidence of renal failure and all-cause mortality. However, due to its inclusion and exclusion criteria, the findings can only be applied to hemodynamically stable outpatients, with eGFR >30 mL/min/1.73m^2^, who received a relatively small amount of contrast media (< 100 mL) (77).

In 2017, Giacoppo and co-workers published the results of a meta-analysis that evaluated data from 124 trials and 28,240 patients investigating different strategies used to prevent CA-AKI. According to their results, fluid administration alone was the least effective preventive strategy for CA-AKI, and in patients with DM none of the strategies used to prevent CA-AKI was effective. Compared to saline administration alone, six other strategies were more effective in reducing the incidence of CA-AKI in that study: statins, N-acetylcysteine, xanthines, sodium bicarbonate, ischemic preconditioning, and the combination of N-acetylcysteine plus sodium bicarbonate (78).

The duration of peri-procedural fluid administration also remains a matter of debate. Previously, patients undergoing coronary angiography were admitted for overnight intravenous fluid administration, and a 12-h isotonic saline administration protocol was applied before and after the procedure. Accumulating data indicate, however, similar results for CA-AKI prevention with 1-h prior and 6-h post-procedural fluid administration (79, 80). The Optimal timing of hydration to erase contrast-associated nephropathy (OTHER CAN) study also failed to show a significant difference in CA-AKI incidence in patients with moderate renal impairment when comparing overnight i.v. to bolus fluid administration (*p* = 0.13) (81). Administration of 250 mL of sodium bicarbonate before contrast administration was also compared with 2,000 mL normal saline administration pre- and post-coronary intervention and was found to be non-inferior (82). In a more recent study, simplified hydration with normal saline from 1 h before to 4 h after coronary angiography at a rate of 3 mL/kg/h was non-inferior to standard hydration with normal saline 12 h before and 12 h after coronary angiography at a rate of 1 mL/kg/h in preventing CA-AKI (83).

### 5.2 Pharmacological agents for prevention of contrast-associated acute kidney injury

Studies on the benefit of natriuretic peptides, aminophylline, theophylline, statins, and ascorbic acid on CA-AKI prevention have yielded mixed results (Table 1). Many other pharmacological agents have also been studied in order to reduce the risk of CA-AKI. Until now, there are inconsistent data about angiotensin converting enzyme inhibitors / angiotensin II receptor blockers, calcium channel blockers, theophylline, ascorbic acid, and statins (84–88). Dopamine, fenoldopam, and atrial natriuretic peptide have shown no benefit, and forced diuresis with mannitol or furosemide is not indicated and may even be dangerous (88). Prostaglandin E1, also known as alprostadil, has been proposed as an effective preventative measure for CA-AKI. A meta-analysis including 19 randomized controlled trials has recently been published, showing that alprostadil might be associated with a reduction in the incidence of CA-AKI. Of the 19 trials, 13 reported the use of prostaglandin E1 to prevent CA-AKI in patients undergoing interventions for coronary heart disease. Even though the results of this meta-analysis showed that the use of prostaglandin E1 might be associated with a significant reduction in CA-AKI, the results require further verification (89).

**Table 1:** Impact of pharmacologic strategies on the occurrence of contrast-associated acute kidney injury.

Beneficial	May be beneficial	Inconsistent data	No benefit	May be harmful
Isotonic saline	Prostaglandin E1 Trimetazidine	Nicorandil Alprostadil *Alpha*-tocopherol Angiotensin converting enzyme inhibitor / angiotensin II receptor blockers Calcium channel blockers Theophylline Ascorbic acid Statins N-acetylcysteine	Dopamine Fenoldopam Atrial natriuretic peptide	Mannitol Furosemide

N-acetylcysteine has been widely used for the prevention of CA-AKI in high-risk patients, mainly because of its favorable side effect profile, low costs, and positive results from some randomized clinical studies. Data are available at present from over 40 clinical trials using N-acetylcysteine for prevention of CA-AKI in high-risk patients, and even though the studies with negative results outnumber those with positive results with a 2:1 ratio, the benefit in the positive studies was significant. The reason for these contradictory results is not yet known but might be related to the use of different types and volumes of contrast agents, different invasive procedures, and different dosages, timings, and routes of N-acetylcysteine administration, or simply to the fact that N-acetylcysteine is not effective in this setting. The first article about the use of N-acetylcysteine to prevent CA-AKI was published by Tepel and co-workers back in 2000. The study included 83 patients undergoing CT with a low-osmolality contrast agent (iopromide). Patients were randomly assigned in two groups: a study group, consisting of 41 patients, who received acetylcysteine (600 mg twice daily) and saline solution (0.45%) intravenously before and after contrast administration, and a control group, consisting of 42 patients, who received placebo and saline. While in the study group only 1 (2%) out of the 41 patients developed CA-AKI, in the control group 9 (21%) out of the 42 patients developed CA-AKI (*p* = 0.01 between groups) (90). In contrast to those results, another randomized controlled trial of N-acetylcysteine to prevent CA-AKI in patients undergoing cardiac angiography published by Durham and co-workers was unable to prove an additional benefit of acetylcysteine along with fluid administration. Seventy-nine patients were enrolled in that study, and there was no significant difference between groups at baseline in any measured parameter. There was no difference between groups in the mean duration of angiography, mean volume of contrast, or mean total intravenous saline administered, and there was also no significant difference between groups in the incidence of CA-AKI. Contrast-associated acute kidney injury occurred in 9 out of 41 patients (22%) in the control group and in 10 out of 38 patients (26.3%) in the group receiving acetylcysteine, with a non-significant statistical difference between the two groups (91). The main differences between Tepel’s and Durham’s trials were the dosage and timing of acetylcysteine administration, the route of administration being the same in both studies. While in Tepel’s trial the patients received acetylcysteine 600 mg orally on the day of administration of the contrast agent and on the day before, on top of intravenous saline administration (90), in Durham’s study the patients received N-acetylcysteine 1,200 mg orally 1 hour prior to and 3 hours following cardiac catheterization (91). Since the elimination half-time of acetylcysteine is 2.1 h and oral administration leads to peak serum levels in approximately 1 hour, the administration in Durham’s trial appears to be more rational from the standpoint of acetylcysteine pharmacokinetics (91). In 2004, Briguori and co-workers emphasized for the first time the potential importance of acetylcysteine dosage. Their study indicated that double dose (i.e., 1,200 mg, orally, twice daily) of N-acetylcysteine seems to be more effective than the standard dose (i.e., 600 mg, orally, twice daily) in preventing CA-AKI, especially in patients receiving high volumes of intra-arterial low-osmolality contrast agents (92). According to the American Society of Nephrology, until a well-powered definitive study will be performed, the use of acetylcysteine is probably reasonable because the drug is safe, well tolerated by patients, and inexpensive. The dose recommended is the dose used by Tepel (i.e., 600 mg twice daily, on the day before and the day of exposure to contrast) (93). An update about CA-AKI from the e-journal of the European Society of Cardiology Council for Cardiology Practice states that the use of acetylcysteine is not contraindicated but does not advocate for its use either (57).

A meta-analysis of 124 trials about preventive strategies for CA-AKI in patients undergoing percutaneous coronary procedures which included N-acetylcysteine, statins, saline, natriuretic peptides, peripheral ischemic preconditioning, ascorbic acid, dopaminergic agents, N-acetylcysteine + sodium bicarbonate, and sodium bicarbonate alone, was published in May 2017. From all the evaluated measures, statin administration was associated with marked and consistent reduction in CA-AKI compared with saline, while the other nine preventive strategies failed to demonstrate any reduction in the incidence of CA-AKI (78). Nicorandil, alprostadil, and *alpha*-tocopherol were also associated with a significant reduction of CA-AKI according to some authors, while others failed to demonstrate the same beneficial effects (94–96). Association of trimetazidine 35 mg twice daily with saline administration 12 h before and after coronary angiography was also shown to reduce the incidence of CA-AKI in some studies (97).

### 5.3 Avoiding potentially nephrotoxic agents

The American College of Radiology Guideline recommended until 2013 temporary discontinuation of metformin in patients undergoing procedures with intravenous administration of contrast media. The American College of Radiology Guideline from 2016 separates these patients into two groups: the group with eGFR ≥30 mL/min/1.73m^2^, in which there is no need to discontinue metformin either prior to or following the administration of contrast media, and the group with eGFR <30 mL/min/1.73m^2^, in which metformin should be temporarily discontinued at the time of or prior to the procedure, withheld for 48 h subsequent to the procedure, and reinstituted only after renal function has been re-evaluated and found to be normal (98).

According to a meta-analysis performed by Wang et al., (99) the use of renin-angiotensin-aldosterone system blockers, including angiotensin-converting enzyme inhibitors and angiotensin II receptor blockers, could be associated with an increased risk of developing CA-AKI. However, larger clinical trials, with more strict inclusion criteria, are needed in order to clarify this hypothesis. Other pharmacological agents, such as certain antibiotics, anticonvulsant, antineoplastic, nonsteroidal anti-inflammatory, hypouricemic, and immunosuppressive agents can also cause acute tubular necrosis, leading to nephrotoxicity and renal impairment, and should thus be avoided in these patients (100).

## 6 Gaps in knowledge and future research

Despite the accumulating data regarding CA-AKI, there are still many unknown aspects related to this condition, particularly in patients with vascular disease, in whom chronic ischemia and inflammation (101) could further contribute to alterations in kidney function. Future studies will have to provide new information regarding the optimal methods of prevention and treatment of this condition following intra-arterial administration of contrast media, which is more and more often encountered in clinical practice. Studies will also have to clarify the long-term impact of CA-AKI on kidney function, to avoid further complications (102). Prophylactic strategies are insufficiently effective, and this is largely due to the fact that the pathophysiology of CA-AKI is not yet sufficiently understood. Further studies will thus have to elucidate CA-AKI pathophysiology and to identify the optimal prophylactic strategies.

The definition of CA-AKI is currently based only on SCr values, but there is no unanimously accepted definition for CA-AKI, and SCr might be too coarse to identify subtle, but potentially relevant changes in renal function following administration of contrast media. Finer markers, such as neutrophil gelatinase-associated lipocalin (NGAL), cystatin-C, interleukin-18, or *beta*-2 microglobulin may be needed to allow earlier and more accurate detection of CA-AKI (103).

Although CA-AKI is considered largely regressive, this conclusion is based only on creatinine monitoring, which is, as mentioned above, a very rough marker. The pathophysiology of CA-AKI, which involves tubular necrosis, suggests that it is very unlikely that contrast agents will have absolutely no impact on the kidney in the long term, at least in certain high-risk patients. Studies using more sensitive markers will have to evaluate this aspect. If a degree of renal damage, even subclinical, persists, this could be relevant if the patient is subsequently exposed to nephrotoxic agents or repeated administration of contrast media. Indeed, in a recent study, we showed, using repeated NGAL evaluation, that acute renal injury was much more common than reflected by SCr, affecting almost 18% of patients undergoing angioplasty procedures. Moreover, our data indicated that unlike CA-AKI, which was regressive at the 1-month follow-up, subclinical kidney injury was still present after 1 month in more than half of patients in whom the kidneys were initially affected by the contrast media (104).

## 7 Conclusion

Contrast-associated acute kidney injury is relatively frequent after intra-arterial administration of contrast agents and has a clinical impact in the short, medium, and long term. Despite the numerous studies carried out, there is still no consensus regarding the predictors and the most effective prophylactic measures for CA-AKI. Overall, data from prospective clinical trials indicate that most prophylactic measures provide negligible impact on CA-AKI. The only exceptions are periprocedural isotonic saline administration and usage of a small volume of contrast media, which have proven their effectiveness in CA-AKI prophylaxis in several large clinical trials. Further studies will have to confirm or deny the protective role of other agents, such as N-acetylcysteine, nicorandil, alprostadil, statins, ascorbic acid, trimetazidine, and others. For intravenous administration of iodinated contrast media, reviewing patients’ medications, optimizing cardiac output, and reducing the doses of contrast agent are efficient means to prevent post-procedural increase in plasma creatinine.

## Author contributions

CS and RP drafted the manuscript. AS supervised the work. All authors contributed to the article and approved the submitted version.
